# Treatment of Typical and Atypical Trigeminal Neuralgia with LINAC-Based Radiosurgery: Complications, Recurrence Rates and Long-Term Treatment Outcomes

**DOI:** 10.3390/jcm15155786

**Published:** 2026-07-24

**Authors:** Marta López-Vicente, Nicolás Cordero-Tous, Carlos Sánchez-Corral, Juan Luis Osorio-Ceballos, Mercedes Zurita-Herrera, José Pablo Martínez-Barbero, Marta Antonia Gómez-González, Gonzalo Olivares-Granados

**Affiliations:** 1 Department of Neurosurgery, Hospital Universitario Virgen de las Nieves, 18014 Granada, Spain; marta.lopez.vicente.sspa@juntadeandalucia.es (M.L.-V.); carlos.sanchez.corral.sspa@juntadeandalucia.es (C.S.-C.); martaa.gomez.sspa@juntadeandalucia.es (M.A.G.-G.); gonzalo.olivares.sspa@juntadeandalucia.es (G.O.-G.); 2 Department of Medical Physics, Hospital Universitario Virgen de las Nieves, 18014 Granada, Spain; juanl.osorio.sspa@juntadeandalucia.es; 3 Department of Radiation Oncology, Hospital Universitario Virgen de las Nieves, 18014 Granada, Spain; mercedes.zurita.sspa@juntadeandalucia.es; 4 Instituto de Investigación Biosanitaria ibs.GRANADA, 18012 Granada, Spain; josep.martinez.sspa@juntadeandalucia.es; 5 Department of Neuroradiology, Hospital Universitario Virgen de las Nieves, 18014 Granada, Spain; 6 Department of Human Anatomy, Universidad de Granada, 18010 Granada, Spain

**Keywords:** radiosurgery, trigeminal neuralgia, BNI scale, neuropathic pain, linear accelerator

## Abstract

**Background/Objectives**: Linear accelerator (LINAC)-based radiosurgery (SRS) is a well-established, non-invasive treatment for pharmacologically refractory trigeminal neuralgia. Despite its widespread use, long-term efficacy and recurrence rates remain incompletely defined, highlighting the need for extended follow-up studies to assess outcome durability. **Methods**: All patients diagnosed with trigeminal neuralgia and treated with LINAC-based SRS (60 Gy, 1–2 mm from the nerve root entry zone) between 2012 and 2019 at a single institution, a national reference center, were recruited for this study. Patients were divided into two categories based on the nature of their neuralgia: typical or atypical. Pain control was assessed using the Barrow Neurological Institute (BNI) scale at 12, 36 and 60 months, as well as time to improvement, recurrence, and adverse effects. **Results**: A total of 112 patients were analyzed, with a mean follow-up period of 105.14 (SD 30.65) months. Statistically significant differences in the success of pain control (BNI I–III) between patients with typical and atypical neuralgia were observed at all evaluated time points (12 months: 87.1% vs. 44.0%, 36 months: 77.0% vs. 22.0%, and 60 months: 60.7% vs. 18.0%) (*p* < 0.001). The mean time to improvement was 6.32 (SD 6.61) weeks (range 0–50), and the recurrence rate following initial improvement was 47.1%. Treatment-related adverse clinical events occurred in 5.4% of patients. **Conclusions**: Long-term pain control is achieved in refractory trigeminal neuralgia cases with LINAC-based SRS, especially in typical cases, with a low incidence of complications.

## 1. Introduction

According to the third edition of the International Classification of Headache Disorders (ICHD-3, 2018), the definition of trigeminal neuralgia according to its diagnostic criteria is as follows: unilateral facial pain occurring as severe recurrent electrical shock-like paroxysms, lasting from a fraction of a second to two minutes, with sudden onset and disappearance, limited to the distribution of one or more branches of the trigeminal nerve division, generally without irradiation, and triggered by innocuous stimuli such as touch, chewing or speech [1]. The etiological framework established by the ICHD-3, 2018, subclassifies trigeminal neuralgia into classical (typically caused by neurovascular compression), secondary (resulting from an underlying structural lesion such as a tumor or multiple sclerosis), and idiopathic forms. Each subtype may present either as purely paroxysmal or accompanied by concomitant continuous pain, although paroxysms remain the defining clinical feature [1]. This classification also distinguishes trigeminal painful neuropathy as a separate entity, characterized by predominantly continuous or near-continuous burning or constrictive pain—often with superimposed paroxysms—and accompanied by sensory abnormalities within the trigeminal distribution, including allodynia and hyperalgesia [1]. Trigeminal neuralgia is a neurological disorder that can be highly disabling and significantly decrease quality of life [1,2]. Despite pharmacological treatment representing the first step in managing this condition, up to 25–50% of patients become refractory or develop intolerance to these types of therapy [3,4,5].

Consequently, trigeminal neuralgia has proven to be a real therapeutic challenge in patients with an inadequate response to pharmacological therapy [2,6]. At this point, different interventional therapeutic options are taken into account, including stereotactic radiosurgery (SRS) among others such as microvascular decompression surgery (MVD), thermocoagulation (TMC), glycerol ablation techniques (GLA) or balloon compression [5]. SRS applied using a linear accelerator (LINAC) has established itself as a minimally invasive, safe and effective technique, providing promising clinical results in terms of pain control and the incidence of adverse effects, with pain relief rates of up to 70–90% in both the short and medium term [7,8,9,10,11]. However, despite published clinical evidence, significant uncertainties remain concerning the long-term outcomes of trigeminal neuralgia after SRS treatment, particularly the lasting effects of pain relief, recurrence rates, and the incidence of neurological complications, in addition to potential variations in response among different clinical subtypes [8].

Against this background, our center is a national referral center for SRS treatment, with a significant number of patients treated and long follow-up periods. Therefore, this study aims to provide solid evidence to optimize the selection of trigeminal neuralgia patients who are candidates for LINAC-based SRS and to more accurately estimate their prognosis and recurrence risk according to clinical subtype.

## 2. Materials and Methods

### 2.1. Project Design and Main Objective

A retrospective observational study was performed based on a systematic review of clinical registries and medical databases from the Hospital Universitario Virgen de las Nieves (Granada, Spain). The information obtained from these sources was subsequently completed and verified through patient interviews performed when participants were contacted to receive information about the study and provide informed consent for participation.

The main objective was to assess the long-term clinical efficacy and safety of LINAC-based SRS as a treatment for trigeminal neuralgia refractory to pharmacological treatment, while reviewing the variations in outcomes between the defined clinical subtypes (typical and atypical trigeminal neuralgia).

### 2.2. Study Population

The study population included all patients over 18 years of age with refractory trigeminal neuralgia who met the inclusion criteria of the Hospital Universitario Virgen de las Nieves’ Trigeminal Neuralgia Protocol to receive SRS with a LINAC between January 2012 and December 2019.

According to this protocol, all patients were initially assessed by the Neurology Department (either at our center or another external facility, due to our hospital’s status as a national referral hospital for SRS treatment). Therefore, a diagnosis of trigeminal neuralgia was made according to the criteria of the International Classification of Headache Disorders applicable at the date of diagnosis, and progressive pharmacological therapy was initiated. High-resolution brain MRI scans, including high-resolution balanced steady-state gradient echo sequences for good differentiation between fluid and tissue, were systematically performed to identify possible etiological agents such as neurovascular compression or structural lesions.

**Figure 1:**
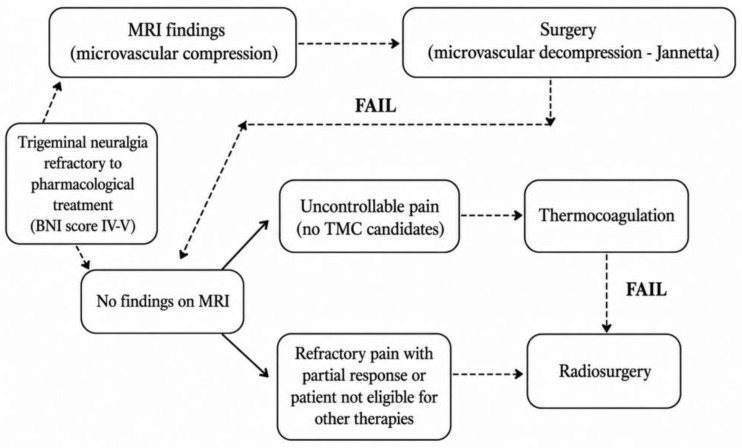
Trigeminal Neuralgia Protocol of Hospital Universitario Virgen de las Nieves, Granada. Abbreviations: BNI: Barrow Neurological Institute, MRI: magnetic resonance imaging, TMC: thermocoagulation.

Referrals were made to the Neurosurgery Department for further evaluation in cases of refractoriness (considered as four or more pharmacological agents used without a clinically significant response), pharmacological intolerance, or relevant radiological findings (Figure 1). Subsequent to this assessment, suitability for SRS treatment was determined by a Multidisciplinary Committee comprising specialists in Neurosurgery, Radiation Oncology, Neuroradiology, and Medical Physics. Once approved by the committee and the patient’s informed consent form was signed, treatment was scheduled.

A total of 119 patients fulfilled the inclusion criteria and were treated during the study period. After identification of all patients with a favorable indication for SRS, only those in whom treatment could not be completed or in whom the clinical documentation essential for evaluating the results was insufficient were excluded from the study. Taking into account these losses before the analysis, 112 patients constituted the evaluable sample. All patients included in the evaluable sample were subsequently contacted and interviewed in order to provide information about the study, complete or verify the clinical follow-up information required for outcome assessment, and obtain informed consent for participation in this project, with no reported losses due to absence of consent. The study was conducted in accordance with the Declaration of Helsinki and approved by the Institutional Review Board (or Ethics Committee) of the Andalusian Research Ethics Committee (code NC-D-02, date of approval: 10 February 2025). This approval referred to the observational study protocol, including retrospective data review, patient interviews, and study-specific informed consent, and not to the original LINAC-based SRS treatment, which had been performed as routine clinical care after treatment-specific informed consent.

### 2.3. Initial Assessment

Demographic and clinical variables were collected for each patient, including age at the time of treatment, categorized age (<55 years, 55–70 years, >70 years), gender, time elapsed between the diagnosis of neuralgia and the application of SRS, and the characteristics and distribution of pain among the branches of the trigeminal nerve. In addition, significant medical history, such as multiple sclerosis (MS) or previous procedures on the trigeminal nerve (TMC, MVD, or SRS), and relevant findings from MRI, in particular the presence of structural lesions or microvascular compression (MVC), were gathered.

**Figure 2:**
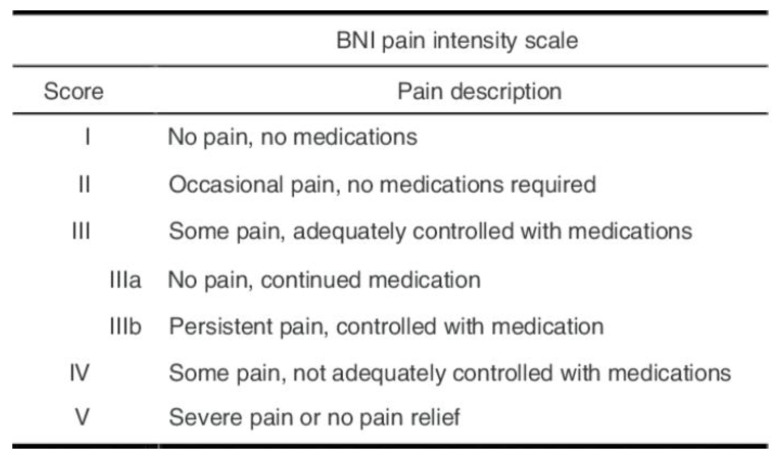
Barrow Neurological Institute (BNI) pain intensity scale for outcome assessment in patients with trigeminal neuralgia. BNI grades I–III were considered to indicate good outcomes, whereas BNI grades IV and V indicate poor response to treatment.

Baseline pain intensity was recorded using the Barrow Neurological Institute (BNI) pain intensity scale (Figure 2), widely established in the literature for evaluating the efficacy of trigeminal neuralgia treatment [11,12,13,14,15].

The literature published prior to the standardization of the ICHD-3, 2018 [1] described two clinical forms of trigeminal neuralgia: typical or type 1 and atypical or type 2 [3,5,16,17]. In order to ensure comparability with previous literature while maintaining current diagnostic accuracy, this study retrospectively subdivided patients into two groups—typical and atypical trigeminal neuralgia—using the following correspondence:

- Purely paroxysmal trigeminal neuralgia, as well as cases with concomitant continuous pain but clinical predominance of paroxysms, regardless of etiology (classical, secondary, or idiopathic); were considered as typical neuralgia.
- Patients with predominantly continuous pain, or pain clinically equivalent to the paroxysmal component, regardless of etiology, were considered as atypical neuralgia [1].

This retrospective classification was considered necessary because the cohort included patients treated over a long period during which clinical terminology was not always uniform. Therefore, all cases were reclassified using a single predefined criterion: the predominant pain phenotype documented before LINAC-based SRS, allowing comparison with previous radiosurgical literature while preserving consistency with the current ICHD-3 diagnostic framework. Because painful trigeminal neuropathy is recognized in the ICHD-3 as a separate entity and may also present with continuous pain, all patients classified within the atypical group were specifically reviewed for features consistent with this diagnosis. None of the patients in the atypical group fulfilled ICHD-3 criteria for painful trigeminal neuropathy. Therefore, the atypical group did not include painful trigeminal neuropathy cases, and no separate neuropathy subgroup analysis was performed.

### 2.4. Stereotactic Radiosurgery: Protocol of Treatment

SRS was administered according to a standardized protocol with uniform technical parameters for all patients. Planning was carried out by a multidisciplinary team that included radiation oncologists, physicists, radiologists, and neurosurgeons. It required the acquisition and fusion of cranial MRI and CT scans obtained under stereotactic conditions, using BrainLab software (iPlan Cranial. Version 3.0, BY, Germany). Treatment was administered with a 6 MeV LINAC using 3 mm or 5 mm circular collimators depending on the individual anatomy. A maximum dose of 60 Gy was prescribed and applied to 1–2 mm from the trigeminal nerve root entry zone in the brainstem (REZ) using 3–10 entry arcs to optimize dosimetric conformation.

### 2.5. Evaluation During Follow-Up

First, the total follow-up time in months after SRS administration was recorded. Pain control was assessed using BNI at defined follow-up points: 12, 36, and 60 months after SRS. As previously reported in the published literature [13,14,15], treatment success was defined as a reduction in pain to BNI categories I–III, while failure was defined as pain corresponding to BNI categories IV–V or if further therapies were needed during the follow-up (Figure 2). Time to improvement was recorded in weeks. Pain recurrence, defined as the return of clinically relevant pain levels (BNI IV–V) or the requirement for additional therapies during the follow-up, was documented in patients who experienced an initial favorable response, whereas the relapse-free interval was recorded in weeks. Likewise, clinical adverse effects associated with the procedure were recorded, and adverse radiological findings resulting from the procedure were analyzed on MRIs performed during follow-up.

### 2.6. Statistical Analysis

A descriptive study was conducted on the frequencies of all variables recorded, including demographic, initial clinical, and follow-up outcome variables. Subsequently, a comparison of proportions was performed using Pearson’s Chi-square (χ^2^) test or Fisher’s exact test when the conditions for the former were not fulfilled for the variables gender, categorized age, history of MS and MVC, type of neuralgia, and previous treatments based on the success recorded at 12, 36 and 60 months. In those comparisons that were statistically significant, the effect was quantified using the absolute difference in proportions, its 95% confidence interval (95%CI) calculated by the Wilson method, and also its significance using the Wald Z-test. In addition, as the BNI scale scores constitute an ordinal variable, the comparison of their distribution between independent groups with statistically significant proportions comparisons was performed using the Kruskal–Wallis test. The evolution of the dichotomous outcome (treatment success versus failure) between the different follow-up points recorded (12, 36, and 60 months) was analyzed using the McNemar test. Kaplan–Meier survival curves were used to represent the time to pain relief following LINAC-based SRS and the time to pain worsening after an initial improvement. Curve comparisons were performed using the Mantel–Cox (log-rank) test, with analyses adjusted for gender, categorized age, type of trigeminal neuralgia (typical vs. atypical), and prior trigeminal procedures. Statistical analysis was performed using IBM SPSS Statistics software, version 29.0 (IBM Corp., Armonk, NY, USA). Results were considered statistically significant for all analyses if *p* < 0.05.

## 3. Results

### 3.1. Descriptive Initial Analysis

A total of 119 patients were included at the time of study closure. Due to seven recorded losses (8.33%), the final sample for analysis consisted of a total of 112 patients. However, during follow-up, one additional patient died after 13 months and was therefore not evaluable for the 36- and 60-month analyses. Among these, 75 patients (66.96%) were referred from other hospitals throughout Spain, after prior assessment by their neurology departments. First, the mean age of the patients included was 67.9 (SD 13.94) years. This variable was categorized for further analysis into three groups: <55 years (19 patients, 17%), 55–70 years (45 patients, 40.2%), and >70 years (48 patients, 42.9%). A descriptive analysis of frequencies was carried out based on the variable type of pain, recording all other demographic characteristics (Table 1).

### 3.2. Analysis of Post-Treatment Outcomes

The mean follow-up time was 105.14 (SD 30.65) months. Results were collected according to the BNI scale at the different points established for the follow-up (12, 36, and 60 months) for both the total sample and, once again, according to the variable type of neuralgia (Table 2).

At 12 months of treatment, a total of 76 treatment success results were recorded (67.9%), 54 (48.2%) of which corresponded to the typical neuralgia group and 22 (19.6%) to the atypical neuralgia group. At 36 months, the absolute number of treatment successes registered was 58 (52.3%), with 47 (42.3%) belonging to the typical neuralgia group and 11 (9.9%) to the atypical neuralgia group. At the last follow-up point (60 months), 45 (40.5%) treatment successes were recorded, 36 (32.4%) of which corresponded to the typical neuralgia group and 9 (8.1%) to the atypical neuralgia group. In addition, the longitudinal evolution of treatment success and failure was analyzed between 12 and 36 months and between 36 and 60 months using the McNemar test, which showed statistically significant changes in both comparisons (*p* < 0.001).

**Figure 3:**
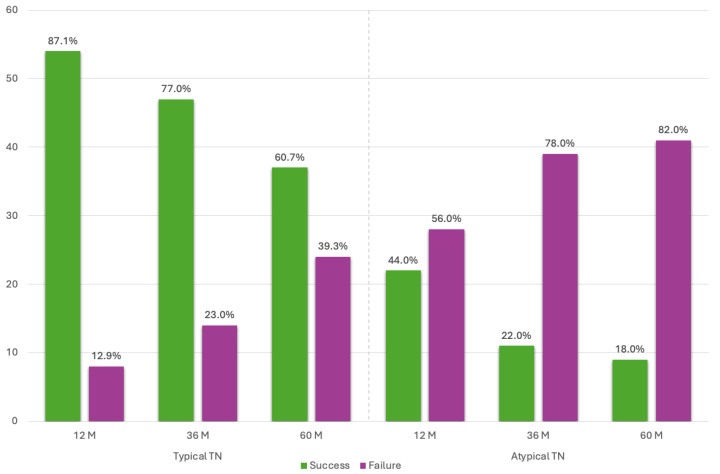
Distribution of treatment results at 12, 36, and 60 months of follow-up according to type of trigeminal neuralgia. Therapeutic success was defined as BNI I–III and failure as BNI IV–V or if additional therapies were necessary before this follow-up point. Percentages above each bar refer to the total number of patients in each subgroup at each follow-up point. Abbreviations: M: months; TN: trigeminal neuralgia.

When treatment outcomes were analyzed within each clinical subtype, patients with typical trigeminal neuralgia showed higher success rates and lower failure rates than those with atypical neuralgia at all follow-up points. The distribution of the treatment success/failure ratio according to the subtype of neuralgia is shown in Figure 3.

Afterwards, in a comparison of proportions using Pearson’s χ^2^ test for the variable type of neuralgia, based on success recorded at 12, 36, and 60 months, the success proportion was statistically significantly higher in typical neuralgia compared to atypical neuralgia at all follow-up points analyzed (*p* < 0.001). The absolute difference in proportions obtained by quantifying the effect of this association was 43.1% (95%CI 25.8–57.4, *p* < 0.001) at 12 months, 55.5% (95%CI 37.2–67.8, *p* < 0.001) at 36 months, and 42.7% (95%CI 24.7–56.6, *p* < 0.001). In addition, Kruskal–Wallis showed statistically significant differences in the distribution of BNI scale scores between typical and atypical neuralgia at all time points analyzed (12, 36 and 60 months: *p* < 0.001), with consistently lower ranks in the typical group.

The remaining analyses comparing proportions by Pearson’s χ^2^ test for the variables gender, categorized age, history of MS and MVC, and previous treatments, again based on success recorded at 12, 36, and 60 months, only found statistically significant differences for previous TMC and SRS. For previous TMC, in all cases, the direction of these statistically significant differences (*p* = 0.002 at 12 months, *p* = 0.011 at 36 months, *p* = 0.047 at 60 months) indicated worse clinical outcomes in patients who had undergone TMC before LINAC-based SRS, with lower success rates compared to those without previous procedures (12 months: 52.9% [27/51 patients] vs. 80.3% [49/61 patients], 36 months: 39.2% [20/51 patients] vs. 63.3% [38/60 patients], 60 months: 31.4% [16/51 patients] vs. 50.0% [30/60 patients]). In addition, the absolute difference in proportions showed a significant 95%CI at all follow-up points analyzed (12 months: 27.4%, 95%CI 9.9–43.0, *p* = 0.002/36 months: 24.1%, 95%CI 5.5–40.6, *p* = 0.011/60 months: 18.6%, 95%CI 0.3–35.1, *p* = 0.047). In contrast, Kruskal–Wallis analysis of the ordinal distribution of BNI also showed statistically significant differences (*p* < 0.01) at all follow-up points (12, 36 and 60 months), with higher mean ranks in the subgroup with prior TMC.

Furthermore, for previous SRS the statistically significant differences were only found at 36 and 60 months (*p* = 0.038 and *p* = 0.006, respectively), with higher success rates for those who had previously received this type of treatment (87.5% [7/8 patients] vs. 49.5% [51/103 patients] at 36 months and 87.5% [7/8 patients] vs. 37.9% [39/103 patients] at 60 months). In the analysis of the absolute difference in proportions, statistically significant differences were also obtained for both analysis points during follow-up (36 months: 38.0%, 95%CI 2.1–51.9, *p* = 0.038/60 months: 49.6%, 95%CI 13.7–63.1, *p* = 0.006). However, analysis of the ordinal distribution of BNI by the Kruskal–Wallis test did not reveal any statistically significant differences between groups.

**Figure 4:**
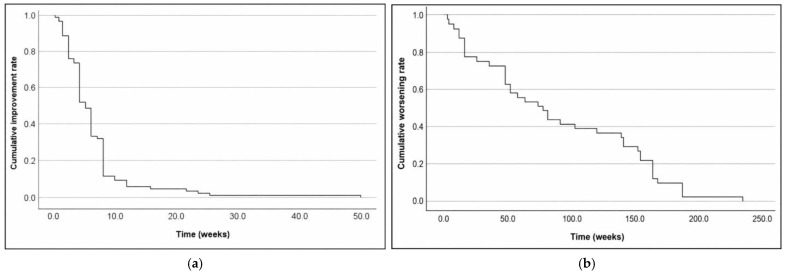
Kaplan–Meier curves representing: (**a**) Time (in weeks) to the onset of pain improvement; (**b**) Time (in weeks) to the onset of worsening after initial improvement.

The next part of the outcome analysis focused on assessing the latency period until improvement after treatment, as well as the recurrence rate and time to recurrence in patients who exhibited initial improvement. The mean time to onset of pain improvement after SRS application was 6.32 (SD 6.61) weeks (range 0–50). The proportion of patients who showed initial improvement was 77.7% (87 patients), although as described above, at the first follow-up point (12 months), the proportion of successes recorded, and therefore of improvement recorded at this point, was 67.9% (76 patients). On the other hand, the proportion of patients who showed initial improvement followed by subsequent worsening was 47.1% (41 patients). The mean time to worsening was 92.2 (SD 67.72) weeks (range 3–239). In addition, Kaplan–Meier curves were used to graphically represent the time to onset of pain relief and the time to onset of worsening after initial improvement (Figure 4a and Figure 4b, respectively).

**Figure 5:**
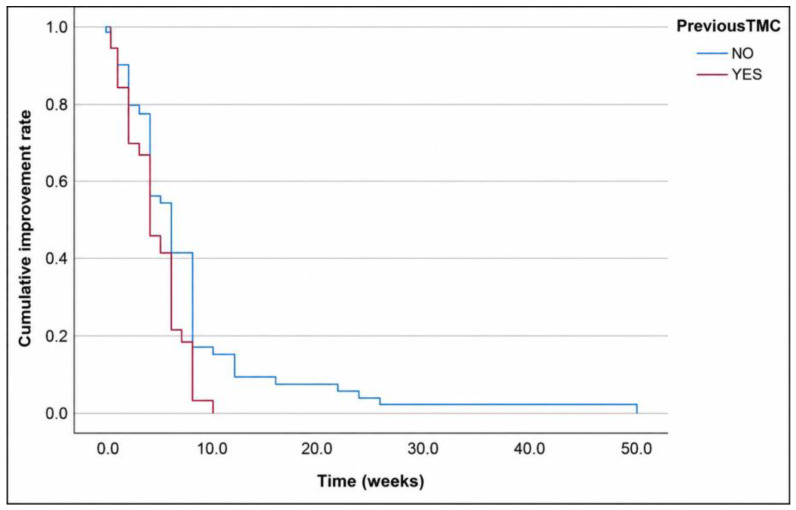
Kaplan–Meier curves representing time to the onset of pain improvement, adjusted for previous TMC.

Second, the Mantel–Cox (log-rank) test was used to compare the Kaplan–Meier curves, adjusted for gender, categorized age, type of trigeminal neuralgia, history of MS, MVC, and previous treatments. Regarding the curves for time to onset of pain improvement, the only variable for which statistically significant differences were found was previous TMC (*p* = 0.012) (Figure 5).

**Figure 6:**
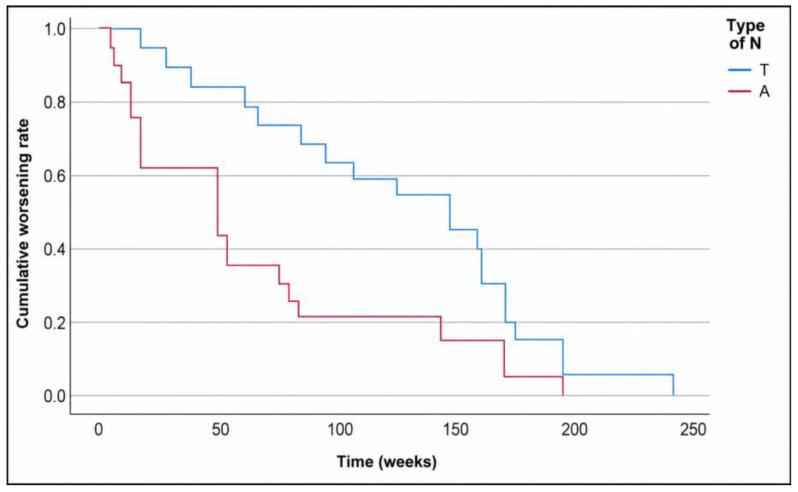
Kaplan–Meier curves representing time to the onset of worsening after initial improvement, adjusted by type of trigeminal neuralgia. Abbreviations: A: atypical neuralgia; N: neuralgia; T: typical neuralgia.

Likewise, for the curves for time to worsening after initial improvement, the only variable for which statistically significant differences were found was the type of neuralgia (*p* = 0.008) (Figure 6).

Finally, both clinical side effects and those detected by MRI during follow-up were analyzed. Sensory alterations were detected in only 6 patients, all of whom had facial hypoesthesia with no impact on their daily quality of life, as no cases of painful anesthesia were recorded in our study. In addition, only one case of radiation necrosis was detected by MRI, but without clinical implications. Accordingly, the rate of clinical side effects in the study was 5.4%, and the rate of side effects detected by MRI was 0.9%. When clinical side effects and MRI-detected side effects were compared according to treatment success at 12, 36, and 60 months using Fisher’s exact test, no statistically significant differences were found.

## 4. Discussion

SRS has established itself as an effective and minimally invasive therapeutic option for trigeminal neuralgia refractory to pharmacological management, particularly in patients who are not candidates for surgery, as well as in those with recurrence after prior procedures [2,6,11]. From its original conception by Leksell in 1951 and the subsequent clinical development of Gamma Knife technology in the second half of the 20th century, this modality has been the historical model for intracranial SRS. This technology established the initial standards of precision, efficacy, and safety in the treatment of neuropathic facial pain [11,18]. Over time, and especially since the 1980s, the adaptation of LINACs for radiosurgical applications has made this technique available to a greater number of institutions, encouraged in part by the presence of these platforms in radiotherapy departments. In recent decades, improvements in immobilization, image-guided verification and dosimetric planning have enabled LINAC-based SRS to achieve levels of precision comparable to those of dedicated systems (such as Gamma Knife). Likewise, the most recent development of frameless strategies in the application of LINAC-based SRS has made it possible to reduce the invasiveness of the procedure while maintaining high levels of spatial accuracy [8,10,19].

Despite these technological advancements, specific evidence on LINAC-based SRS for trigeminal neuralgia remains more limited and heterogeneous than that available for Gamma Knife, especially in relation to long-term follow-up studies and the systematic identification of clinical and therapeutic prognostic factors [11,13,19]. Consistent with the ongoing technological evolution of SRS, recent series, such as that published by Shields et al. in 2024 [8], have reported encouraging clinical outcomes using frameless techniques. These results highlight the need for large, well-characterized cohorts to consolidate the role of LINAC within the current therapeutic algorithm. Rather than representing a directly comparable technical approach, the present study is aligned with this line of research. This work reports a LINAC frame-based SRS series with long-term follow-up and a detailed assessment of the impact of clinical subtypes and previous treatments on the response to SRS. In addition, our sample stems from the activity of a national reference center for SRS, which provides treatment to numerous patients referred from different centers. Consequently, it constitutes a large and representative sample of trigeminal neuralgia refractory to pharmacological therapy treated within a standardized multidisciplinary approach.

From a demographic and clinical perspective, the baseline features of our sample are broadly comparable to those described in the main series on SRS for trigeminal neuralgia. The advanced age of the patients treated, the predominance of women, the distribution of affected trigeminal branches, and the high proportion of patients with severe baseline pain, predominantly in BNI categories IV–V, reflect the clinical profile typically referred for radiosurgical assessment [2,6,11,13]. Although a small proportion of patients had BNI I–III prior to treatment, this finding should be interpreted in the context of the fluctuating nature of trigeminal neuralgia, the possible partial or transient response to previous medication or procedures, and intolerance to pharmacological treatment at effective doses [12,20]. Overall, these baseline characteristics reinforce the external validity of our study and provide an appropriate clinical framework for comparison with published radiosurgical series.

Regarding overall efficacy, the results observed in our series fall within the spectrum described in the contemporary literature on LINAC-based SRS using stereotactic frame techniques, characterized by significant initial clinical improvement followed by a progressive decrease in pain control throughout follow-up. Current long-term follow-up studies have reported clinically significant pain relief rates during the first year, usually around 65–85%, followed by a gradual decline in clinical benefit over the follow-up period [11,13,21,22]. In line with this temporal pattern, our previous series reported a decline in clinical improvement from 68.11% at 12 months to 58.21% at 36 months [13]. Debono et al., in one of the largest series of LINAC dedicated/frame-based SRS with long-term follow-up, reported an observed initial response of 90.7% and estimated, through actuarial analysis, a probability of maintaining pain relief of 85.0% at 1 year, 65.8% at 5 years and 48.1% at 10 years [21]. Consistent with these observations, pain control (BNI I–III) at 12 months in our sample was 67.9%, gradually dropping to 52.3% at 36 months and 40.5% at 60 months of follow-up. This decline was also supported by the longitudinal analysis of treatment success and failure using the McNemar test, which showed statistically significant changes between 12 and 36 months and between 36 and 60 months. This subsequent evolution during extended follow-up is consistent with the temporal pattern described in the LINAC-based radiosurgical literature, reinforcing the comparability of our medium- and long-term results.

Nevertheless, a comparative assessment of the clinical efficacy results requires consideration of the technical heterogeneity that exists among published series on LINAC-based SRS for trigeminal neuralgia. These disparities can be broadly grouped into three main domains: immobilization strategy, radiosurgical platform, and treatment-planning parameters. Examples of immobilization strategies include stereotactic frames, thermoplastic masks, and image-guided frameless techniques. Radiosurgical platforms range from conventional LINACs adapted for SRS to dedicated LINAC systems and robotic systems such as CyberKnife. Finally, planning parameters may vary in terms of prescribed dose, collimator size, spatial verification, irradiated nerve volume, and anatomical target location [7,8,9,10,11,13,21,22,23,24,25].

From the perspective of immobilization, the study published by Kienzler et al. in 2022 [10], provides one of the most significant current comparisons of LINAC frame-based versus mask-based SRS for trigeminal neuralgia. In their cohort of 234 patients, including 137 treated with a frame-based technique and 97 with a mask-based technique, the frame-based group achieved higher adequate pain control (BNI I–III) both initially (93.4% vs. 87.6%) and at ≥24 months (89.9% vs. 77.8%), as well as a lower recurrence rate (20.4% vs. 38.1%). As background, Chen et al. [25] reported no significant differences in pain relief in a smaller 2015 comparison of 34 patients treated with rigid frame and 14 patients treated with facemask/frameless image-guided SRS. However, the authors noted potential procedural advantages of frameless techniques by avoiding rigid cranial fixation. More recently, Shields et al. [8] in 2024 reported encouraging results in a cohort of 116 patients treated with frameless LINAC SRS, with a mean follow-up of 44.1 months and a BNI I–III at the last follow-up in 94.8% of cases. Additional evidence from frameless SRS series, including image-guided LINAC and robotic platforms such as CyberKnife, further supports the clinical potential of these approaches [8,9,21,23,24]. However, because most of this evidence derives from non-comparative cohorts, these results should not be interpreted as directly equivalent to LINAC frame-based series. Overall, these findings suggest that frameless strategies, including mask-based, image-guided frameless, and robotic approaches, may improve procedural tolerability and patient comfort. Nevertheless, LINAC frame-based SRS remains a precise technique with robust and durable outcomes in the available comparative evidence.

Beyond immobilization, dose prescription and anatomical target definition represent additional sources of variability across radiosurgical series. Most published studies have used maximum doses in the range of 70–90 Gy, usually targeting the REZ or more anterior cisternal or retrogasserian segments, without a definitive consensus regarding the optimal dose or target [11,13,22,26,27]. In the present series, consistent with the institutional experience previously reported, all patients were treated using a uniform institutional protocol of 60 Gy prescribed 1–2 mm from the REZ, using 3 mm or 5 mm circular collimators according to individual anatomy [13]. This strategy differs from other radiosurgical series for trigeminal neuralgia, including both LINAC and Gamma Knife, in which higher doses, different collimator sizes, longer irradiated nerve segments, or more anterior/retrogasserian targets have been used [7,8,9,21,22,23,24,26,27,28]. Therefore, differences in dose and target must be considered alongside immobilization and the radiosurgical platform used when interpreting the variability in efficacy, recurrence, and sensory toxicity between series. Within this heterogeneous technical landscape, the standardized protocol used in the present study provides a consistent framework for interpreting our outcomes.

In summary, our global efficacy results show qualitative and quantitative agreement with the clinical evolution described after LINAC-based SRS and, more broadly, with the experience accumulated with Gamma Knife, whose long-term outcomes remain within comparable ranges of sustained pain control [11,18,28,29,30]. In a recent long-term Gamma Knife series, Sato et al. [28] evaluated 103 patients with at least 10 years of follow-up and reported adequate pain relief, defined as BNI I–IIIa, in 82.5% of patients initially and in 58.2% at final follow-up. Although direct equivalence between platforms is limited by the same technical heterogeneity previously discussed for LINAC-based modalities, these data help to contextualize our findings within the expected range of radiosurgical outcomes for trigeminal neuralgia. Against this background, the main contribution of our study lies in providing long-term results obtained from a homogeneous cohort of patients treated with LINAC frame-based SRS, allowing clinical outcomes and prognostic factors to be interpreted with less interference from internal technical variability. A detailed comparison between the main radiosurgical series cited and our findings is summarized in Table 3, facilitating an integrated interpretation of initial efficacy and durability over time.

Apart from the overall efficacy results, one of the most clinically relevant findings of our study was the significant influence of the clinical phenotype on the response and durability of pain control following LINAC frame-based SRS. This comparison should be interpreted taking into account that the distinction between typical and atypical neuralgia was made to ensure comparability with previous radiosurgical literature, adapting the historical concepts of ‘typical/type 1’ and ‘atypical/type 2’ to the predominant pain phenotype according to the current classification of the ICHD-3, 2018 [1]. Accordingly, purely paroxysmal forms or those with a clear paroxysmal predominance were considered typical, and those with predominantly continuous pain or pain equivalent to paroxysmal, were considered atypical. Based on this classification, in a sample of 62 patients classified as presenting typical neuralgia and 50 as atypical neuralgia, the typical group showed significantly higher rates of adequate pain control—BNI I–III—at all follow-up points assessed: 87.1% versus 44.0% at 12 months, 77.0% versus 22.0% at 36 months, and 60.7% versus 18.0% at 60 months, all of which were statistically significant. The magnitude of this difference was also significant, with absolute differences in proportions of 43.1%, 55.5% and 42.7% at 12, 36 and 60 months, respectively, all of which were statistically significant. Furthermore, this superiority was not limited to a higher proportion of patients classified as achieving therapeutic success, but was also reflected in a better overall pain control profile. In the ordinal analysis of the BNI scale scores, residual pain intensity was lower in the typical group throughout the follow-up period. Finally, Kaplan–Meier analysis showed that clinical subtype was the only variable significantly associated with time to worsening following initial improvement, with a later recurrence observed in patients with typical neuralgia. Overall, these results suggest that the predominant pain phenotype serves not only as a descriptive category, but also as a relevant prognostic factor for estimating the efficacy and durability of response after SRS. From a clinical perspective, the magnitude of these differences may help to improve patient selection, pre-treatment counseling and the management of expectations. Patients with typical trigeminal neuralgia appear to constitute the subgroup most likely to achieve sustained pain control following LINAC-based SRS. This supports SRS as a particularly reasonable option for this clinical phenotype when pharmacological treatment is ineffective or poorly tolerated. Conversely, patients with atypical phenotypes should not necessarily be excluded from SRS, but the indication should be assessed on a case-by-case basis, with more cautious expectations regarding the probability and duration of the response and taking into account alternative or complementary therapeutic strategies. Importantly, this difference should not be interpreted as being explained by the formal inclusion of painful trigeminal neuropathy cases, as no patient in the atypical group fulfilled strict ICHD-3 criteria for this diagnosis.

This differential pattern based on clinical phenotype does not appear to be an isolated finding in our study group, since it is supported by previous radiosurgical literature. Nevertheless, studies that stratify results by clinical subtype are limited and use definitions that are not always consistent [8,13,30,31,32,33,34]. Within the specific field of LINAC-based SRS, in our previous institutional study typical trigeminal neuralgia was associated with better outcomes than atypical neuralgia. Response rates were higher in the typical group at both 12 months, 77.08% versus 47.61%; and 36 months, 53.19% versus 30.0%, with statistically significant differences between the two clinical subtypes [13]. Beyond this series, the evidence from LINAC studies that analyze outcomes according to clinical phenotype remains limited and methodologically heterogeneous [7,8,31]. By contrast, the literature on Gamma Knife provides more extensive and clinically consistent evidence regarding the influence of the neuralgia phenotype on the radiosurgical response. Dhople et al. [30] provide an indirect comparative framework between atypical and classic trigeminal neuralgia. In their series of 35 patients with atypical neuralgia, 72% had a favorable clinical outcome, with a mean time to relief of 5.8 weeks and a mean duration of benefit of 62 weeks [33]. In the same group’s experience with typical neuralgia, 81% of 95 patients achieved BNI I–III, with a median latency of 2 weeks and a median duration of response of 32 months. Even though the two series do not allow for a direct, homogeneous comparison, these data suggest that atypical neuralgia may respond to SRS, although with a potentially reduced, slower and shorter-lasting response. More recently, Dong et al. [32], in a series of 158 patients treated with Gamma Knife—46 with concomitant continuous pain and 112 without this component—observed that the long-term rate of complete pain relief was significantly lower in patients with concomitant continuous pain, 15.2% versus 35.7%. Collectively and consistent with our findings, these reports suggest that the presence of a persistent or atypical pain component is associated with a shorter-lasting and/or reduced radiosurgical response.

In addition to the clinical phenotype, the history of previous trigeminal nerve interventions provided further insight into the complexity of the disease course and the subsequent response to SRS. In our series, prior TMC was significantly associated with lower success rates at 12, 36 and 60 months. Moreover, ordinal analysis of the BNI scale scores indicated that this effect was not limited to a lower probability of achieving therapeutic success but significantly reflected worse overall pain control during follow-up. Direct comparison with the literature is limited, as most studies group various prior procedures under broad categories of ablative treatment, without consistently isolating the specific effect of TMC [7,11,19]. Nevertheless, our results are consistent with the study by Marshall et al., in which prior radiofrequency ablation was identified as an independent factor associated with reduced efficacy following Gamma Knife SRS [35]. Combined, these findings suggest that prior TMC could identify a subgroup with increased clinical resistance and a lower probability of a long-term radiosurgical response.

On the other hand, a history of previous SRS adds a further dimension to previous trigeminal treatments, as its impact appeared to be concentrated on the probability of maintaining a favorable medium- and long-term response. In our series, no significant differences were observed at 12 months; however, patients previously treated with SRS showed higher absolute rates of therapeutic success at 36 and 60 months. Given that this subgroup included only eight patients, this finding should be interpreted as exploratory and underpowered despite the statistical significance observed in the subgroup analysis. The literature on repeat SRS helps to put this finding into context: although a second SRS may provide clinical relief, the response appears to depend largely on the outcome of the first treatment. In the meta-analysis recently published by Valeri et al., which included 461 patients who underwent a second SRS, 73% achieved BNI ≤ III following retreatment, but 31.9% of responders experienced subsequent recurrence. In addition, a favorable response to the first SRS was associated with a six-fold higher probability of pain control following repeat treatment [36]. This may provide a plausible explanation for the direction of our results, since patients selected for a repeat SRS may constitute a clinically selected subgroup, possibly including patients who had responded to the first treatment and subsequently relapsed. However, given that the clinical response to the initial SRS was not systematically recorded in our sample, this interpretation should be regarded as hypothesis-generating rather than confirmatory. Within this framework, our findings suggest that prior SRS should not be interpreted as a uniform prognostic factor, but rather as an indicator of a clinically heterogeneous retreatment context. The literature suggests that the response to the initial treatment and careful patient selection may determine the benefit of a further SRS.

In contrast, prior MVD showed no significant association with the response in our series, suggesting that not all previous trigeminal interventions carry the same prognostic significance. This finding is consistent with the general literature on SRS. Although the systematic review by Tuleasca et al. [11] highlights the methodological heterogeneity in the available series, prior MVD has not been consistently identified as a factor that negatively affects the efficacy of SRS. Similarly, several individual studies have also failed to identify any clear adverse effect of prior surgery on radiosurgical outcomes, including our previous institutional experience [7,13,19]. Furthermore, studies focusing on patients treated with SRS following prior MVD, such as those published by Kano et al. and Horn et al., have confirmed that SRS can maintain significant clinical utility, with favorable response rates close to 80–85% [37,38]. This distinction is clinically significant, as MVD is a decompressive, non-ablative procedure and should therefore not be considered in conjunction with percutaneous ablative techniques or SRS. In summary, these results suggest that previous trigeminal procedures should not be interpreted as a homogeneous category, but rather according to their mechanism and clinical context. This interpretation qualifies the previous institutional experience [13], where the typical phenotype was the main prognostic factor, and suggests that, with a larger sample size and prolonged follow-up, specific prior procedures such as TMC and SRS may become relevant to the durability of pain control. Therefore, future studies should analyze these procedures separately, avoiding the grouping of techniques with different mechanisms of action and potentially different effects on the trigeminal nerve.

Furthermore, the response following SRS showed a clearly time-dependent pattern in our series. Improvement occurred after a mean of 6.32 weeks and was initially observed in 77.7% of patients, although 47.1% of responders subsequently experienced a worsening of symptoms, with a mean time to deterioration of 92.2 weeks. This pattern is consistent with our previous institutional experience and with the existing literature on SRS involving long-term follow-up, in which an initial response is usually frequent, but the duration of the benefit gradually decreases over time [10,11,13,30]. However, comparisons between studies should be interpreted with caution, due to the considerable variability in definitions of response, duration of follow-up, radiosurgical techniques and baseline patient characteristics. This heterogeneity is reflected in the systematic review by Tuleasca et al., in which reported recurrence rates ranged from 0 to 52.2% for Gamma Knife and 19–63% for LINAC [11]. Therefore, the efficacy of SRS should not be interpreted exclusively in terms of success rates at fixed time points, but rather as a dynamic process in which the time to benefit and its duration represent complementary dimensions of the therapeutic response. From a pathophysiological perspective, the clinical pattern observed—initial response followed by progressive recurrences—is consistent with the biological mechanisms proposed for the effect of SRS on the trigeminal sensory root. These mechanisms include selective axonal damage, focal demyelination, and altered ectopic transmission in afferent fibers [29,39,40,41]. These processes explain both the latency in the onset of pain relief after SRS and the possible loss of long-term efficacy associated with neural reorganization or partial regeneration of irradiated tissue [42].

Assessing the time to improvement and the time to worsening also made it possible to distinguish between factors related to the latency of the response and factors linked to the duration of the benefit. In our series, previous TMC was the only factor significantly associated with a shorter time to improvement, a finding that might seem paradoxical when considered alongside their lower success rates during the follow-up period. Although the specific impact of preceding TMC on response latency following SRS does not appear to have been systematically evaluated in the available radiosurgical literature, this finding supports a plausible pathophysiological interpretation. Percutaneous radiofrequency procedures produce a controlled thermal lesion on the trigeminal nerve, with the aim of modifying nociceptive transmission [43], whereas SRS has been linked to delayed microstructural changes in the trigeminal root, as previously described [29,39,40,41]. In a nerve structure that has previously been damaged or functionally altered by TMC, the additional radiosurgical effect may reach the threshold required to produce clinical relief more quickly. However, this shorter latency should not be interpreted as an indicator of a consistently better prognosis, as these patients probably represent a more refractory subgroup with a more complex neuropathic condition. Conversely, the duration of the benefit appeared to depend primarily on the clinical phenotype in our series. The type of neuralgia was the only factor significantly associated with the time to worsening after initial improvement, with a more sustained course observed in patients with typical neuralgia. Although most studies have focused on response rates at fixed follow-up points rather than on the durability of pain relief according to clinical phenotype, our findings are consistent with previous studies on SRS. This reinforces the fact that the atypical or persistent component of pain not only reduces the overall probability of response, but also compromises the stability of the benefit once it has been achieved [32,33].

Finally, the safety profile observed in our series reinforces the role of LINAC-based SRS as a minimally invasive option for patients with refractory trigeminal neuralgia. Treatment-related clinical adverse effects occurred in only 5.4% of patients, all in the form of facial hypoesthesia, with no impact on quality of life and no cases of painful anesthesia. Furthermore, only one radiological finding consistent with radionecrosis (0.9%) was detected, with no clinical implications. The absence of a significant association between the occurrence of adverse effects and therapeutic success at 12, 36 and 60 months also suggests that clinical benefit was not dependent on the development of significant toxicity. This safety profile is consistent with the previous radiosurgical literature, in which sensory disturbances constitute the most frequent adverse effect, while serious complications, such as painful anesthesia or symptomatic radiological injury, are rare. However, published rates vary according to dose, target location, technique used, duration of follow-up and definition of toxicity [8,10,11,13,19]. Taken together, these data reinforce the risk-benefit balance of LINAC-based SRS, particularly in patients with refractory disease or with reduced tolerance to more invasive procedures.

### Limitations

The main limitation of this study stems from its retrospective, single-center design, which may reduce the level of evidence, limit control over potential confounding factors, and increase the risk of bias inherent in the retrospective collection of clinical data. However, this limitation is partially mitigated by the systematic review of clinical records and the follow-up interviews with all patients included in the evaluable sample to complete or verify clinical information. Additional factors that help offset this limitation include the relatively large sample size for a LINAC series, the extended follow-up period, the application of a standardized technical protocol, and the fact that the study was conducted at a national referral center with systematic multidisciplinary assessment. Another relevant limitation is that the comparison with the available literature is made in a field where much of the evidence comes from series treated with Gamma Knife, while specific data on LINAC-based SRS are more limited and technically heterogeneous. Therefore, comparisons between studies should be interpreted with caution. Furthermore, the classification into typical and atypical neuralgia was carried out retrospectively, adapting historical categories used in the radiosurgical literature to the current clinical classification. Although this approach allows for comparability with previous studies, it may introduce a certain degree of heterogeneity and potential classification bias. Finally, the clinical heterogeneity of the patients and the presence of small subgroups—particularly in relation to prior SRS—may reduce the precision of some estimates and limit the statistical power of subgroup analyses. Therefore, these findings should be interpreted cautiously and considered exploratory. Nevertheless, the combination of long-term follow-up, technical homogeneity, and longitudinal assessment of response provides valuable information for contextualizing the efficacy, durability, and safety of LINAC-based SRS in patients with refractory trigeminal neuralgia.

## 5. Conclusions

LINAC-based SRS represents an effective and safe treatment option for refractory trigeminal neuralgia, although there is a progressive loss of benefit during long-term follow-up. The clinical phenotype appears to be an important factor in prognostic stratification: typical neuralgia is associated with a higher probability of pain control and a longer-lasting benefit, while atypical or persistent features in pain identify patients at higher risk of a less favorable response and reduced benefit duration. Previous trigeminal procedures should also be interpreted with caution, as they may identify subgroups of greater therapeutic complexity rather than a single homogeneous prognostic category. The favorable safety profile observed reinforces the risk-benefit balance of LINAC-based SRS, particularly in patients with refractory disease or with lower tolerance to more invasive procedures. Future prospective, multicenter studies should validate these findings and analyze, in a differentiated approach, the impact of the clinical phenotype and of each previous procedure on the latency, durability and safety of the radiosurgical response.

## Figures and Tables

**Table 1:** Distribution of demographic characteristics according to type of neuralgia (typical and atypical). Abbreviations BNI: Barrow Neurological Institute; F: female; M: male; MS: multiple sclerosis; MVC: microvascular compression; MVD: microvascular decompression (Jannetta); Me: median number of months; n: absolute frequency; p: percentage of the total (112 patients); r: range; SRS: radiosurgery; TN: trigeminal neuralgia; TMC: thermocoagulation; y: years.

	Typical TN 62 (55.4%)	Atypical TN 50 (44.6%)
**Gender; n (p)**		
*m*	23 (20.5)	22 (19.6)
*f*	39 (34.8)	28 (25.0)
**Age (y); Me (r)**	70 (43–97)	64.5 (32–98)
**Disease time; Me (r)**	120 (12–480)	60 (9–408)
**Medical History; n (p)**		
MS	2 (1.8)	11 (9.8%)
MVC	16 (14.3)	7 (6.3)
**Affected branches; n (p)**		
V1	0 (0.0)	0 (0.0)
V2	16 (14.3)	3 (2.7)
V3	14 (12.5)	13 (11.6)
2 branches	26 (23.2)	24 (21.4)
All	6 (5.4)	10 (8.9)
**Previous treatments; n (p)**		
TMC	21 (18.8)	30 (26.8)
MVD	8 (7.1)	9 (8.0)
SRS	6 (5.4)	2 (1.8)
**Initial BNI scale score; n (p)**		
I–III	5 (4.5)	3 (2.6)
IV–V	57 (50.9)	47 (42.0)

**Table 2:** Distribution of BNI scale scores at 12, 36, and 60 months of treatment for both the total sample (the denominator was 112 patients at 12 months and 111 patients at 36 and 60 months because one patient died after 13 months of follow-up) and according to the type of neuralgia variable (typical and atypical neuralgia). Abbreviations: BNI: Barrow Neurological Institute Scale; n: absolute frequency; p: percentage calculated using the denominator available at each follow-up point; OTB: other therapies needed before the point of follow-up; TN: trigeminal neuralgia.

	Typical TN: 62 (55.4%)	Atypical TN: 50 (44.6%)	Total: 12 M: 112 (100%) 36–60 M: 111 (100%)
**BNI 12 M; n (p)**			
I	3 (2.7)	1 (0.9)	4 (3.6)
II	0 (0.0)	0 (0.0)	0 (0.0)
IIIa	18 (16.1)	6 (5.4)	24 (21.5)
IIIb	33 (29.5)	15 (13.4)	48 (42.9)
IV	3 (2.7)	8 (7.1)	11 (9.8)
V	3 (2.7)	14 (12.5)	17 (15.2)
OTB	2 (1.8)	6 (5.4)	8 (7.1)
**BNI 36 M; n (p)**			
I	8 (7.2)	0 (0.0)	8 (7.2)
II	3 (2.7)	0 (0.0)	3 (2.7)
IIIa	17 (15.3)	7 (6.3)	24 (21.6)
IIIb	19 (17.1)	4 (3.6)	23 (20.7)
IV	2 (1.8)	10 (9.0)	12 (10.8)
V	1 (0.9)	9 (8.0)	10 (8.9)
OTB	11 (9.9)	20 (18.0)	31 (27.9)
**BNI 60 M; n (p)**			
I	12 (10.8)	0 (0.0)	12 (10.8)
II	0 (0.0)	0 (0.0)	0 (0.0)
IIIa	16 (14.4)	6 (5.4)	22 (19.8)
IIIb	8 (7.2)	3 (2.7)	11 (9.9)
IV	4 (3.6)	9 (8.1)	13 (11.7)
V	1 (0.9)	7 (6.3)	8 (7.2)
OTB	20 (18.0)	25 (22.5)	45 (40.5)

**Table 3:** Comparative summary of the main radiosurgical series for trigeminal neuralgia and the present study, divided according to radiosurgical modality and including analyzed groups, target, prescribed dose, and main pain-control outcomes. Abbreviations: BNI: Barrow Neurological Institute; CCP: concomitant continuous pain; CKRS: CyberKnife radiosurgery; FFP: freedom from pain; FU: follow-up; GKRS: Gamma Knife radiosurgery; Gy: gray; LINAC: linear accelerator; M: months; MS: multiple sclerosis; n: number; REZ: trigeminal nerve root entry zone; SRS: stereotactic radiosurgery; TN: trigeminal neuralgia.

Author	n/Analyzed Groups	Target	Dose	Pain-Control Outcomes
**LINAC-based SRS series**				
Debono et al., 2019 [21]	301 classic TN	Anterior retrogasserian/cisternal target	90 Gy	Initially pain-free: 90.7%. BNI I–IIIa: 85.0%, 76.1%, 65.8% and 48.1% at 1/2/5/10 years.
Kienzler et al., 2022 [10]	234: 137 frame-based/97 mask-based	REZ	90 Gy	BNI I–III frame vs. mask: 93.4% vs. 87.6% initially; 89.9% vs. 77.8% at ≥24 M.
Smith et al., 2011 [22]	169 analyzed/179 treated; three dose/isodose cohorts	REZ	70–90 Gy	Significant pain relief: 79.3%; by regimen: 64.0%, 79.0% and 88.0%.
Shields et al., 2024 [8]	116, all BNI IV–V pre-treatment	Meckel’s cave/dorsal REZ	80/85/90 Gy; mean 85.6 Gy	BNI I–III at last FU: 94.8%.
Cordero Tous et al., 2017 [13]	71: 48 typical TN/23 atypical TN	1–2 mm from the REZ	60 Gy	Pain improvement: 68.1% at 12 M and 58.2% at 36 M; typical vs. atypical TN: 77.1% vs. 47.6% at 12 M and 53.2% vs. 30.0% at 36 M.
López-Vicente et al., present study	112 analyzed: 62 typical TN/50 atypical TN	1–2 mm from the REZ	60 Gy	BNI I–III: 67.9%, 52.3% and 40.5% at 12/36/60 M; typical vs. atypical TN: 87.1% vs. 44.0%, 77.0% vs. 22.0% and 60.7% vs. 18.0% at 12/36/60 M.
Rashid et al., 2018 [7]	55: 37 classic, 15 MS-related, 2 symptomatic and 1 atypical TN	REZ or retrogasserian target	90 Gy	BNI I–IIIa: 69% overall and 88.8% in classic TN at last FU.
Chen et al., 2010 [25]	44 typical TN	Trigeminal nerve in the ambient cistern	90 Gy	Satisfactory pain relief: 91%; 12-M actuarial freedom from recurrence: 78%.
Kundu et al., 2022 [19]	32 analyzed/41 treated	REZ	80–90 Gy, mostly 90 Gy	BNI I–III: 72%.
Pokhrel et al., 2017 [31]	27: 22 typical TN/5 atypical TN	Cisternal trigeminal nerve root	80 Gy	Typical TN: pain relief 82%; atypical TN: no response.
**Cyber Knife series**				
Guillemette et al., 2022 [24]	166 patients/168 procedures	Cisternal trigeminal nerve	Median maximum dose 80 Gy	Adequate pain relief: 86.9%. Actuarial maintenance of pain relief: 77%, 63% and 50% at 1/3/5 years.
Stergioula et al., 2024 [23]	50 refractory TN	Elongated cisternal trigeminal nerve segment	Median 60 Gy	BNI I–III: 74%. Actuarial FFP: 82%, 78% and 74% at 24/36/>48 M.
**Gamma Knife series**				
Régis et al., 2016 [18]	497 classical TN	Anterior retrogasserian target	Median 85 Gy	Initially pain-free: 91.8%. Pain-free without medication: 71.8%, 64.9%, 59.7% and 45.3% at 3/5/7/10 years.
Dong et al., 2024 [32]	158: 46 with CCP/112 without CCP	REZ	70–90 Gy central dose	Initial excellent/good outcome: 86.1%. Long-term BNI I–IIIa: 62.0%; complete pain relief 15.2% with CCP vs. 35.7% without CCP.
Sato et al., 2023 [28]	103 analyzed from 249 treated	Retrogasserian target	90 Gy	Adequate pain relief: 82.5% initially and 58.2% at final FU.
Dhople et al., 2009 [30]	95 classic TN analyzed/112 treated	Adjacent to REZ	Median 75 Gy	Initial BNI I–III response: 81%. Treatment failure-free rate: 60%, 41%, 34% and 22% at 1/3/5/7 years.
Dhople et al., 2007 [33]	35 atypical TN treated; 32 with FU	Adjacent to REZ	Median 75 Gy	Excellent/good outcome: 72%; actuarial recurrence-free rate: 74%, 54% and 54% at 1/2/3 years.
**Systematic reviews**				
Tuleasca et al., 2019 [11]	6461 patients/65 studies	Mainly REZ; anterior cisternal/retrogasserian targets also reported	GKRS 60–97 Gy; LINAC SRS 50–90 Gy; CKRS 66–90 Gy	Initial FFP without medication: GKRS 53.1%, LINAC SRS 49.3%, CKRS 56.3%.
De La Peña et al., 2022 [9]	1705 patients/30 studies; institutional series n = 23	REZ or cisternal segment	Institutional median 85 Gy; review median 75 Gy	Institutional BNI I–III: 83%. Pooled excellent/good response: 63.1%/16.1%.

## Data Availability

The data supporting the findings of this study are not publicly available due to ethical and privacy restrictions relating to the use of individual patient clinical data. Anonymized data may be made available upon reasonable request to the corresponding author, provided that such a request is approved by the respective ethics committee and complies with applicable institutional regulations.

## Abbreviations

The following abbreviations are used in this manuscript:

BNI	Barrow Neurological Institute
CI	Confidence Interval
CT	Computed Tomography
Gy	Gray
GLA	Glycerol ablation techniques
ICHD-3	International Classification of Headache Disorders, 3rd edition
LINAC	Linear Accelerator
MeV	Megaelectronvolt
MRI	Magnetic Resonance Imaging
MS	Multiple Sclerosis
MVC	Microvascular compression
MVD	Microvascular decompression (Jannetta)
REZ	Trigeminal nerve root entry zone
SRS	Stereotactic radiosurgery
TMC	Thermocoagulation
